# Improving Information Extraction from Pathology Reports using Named Entity Recognition

**DOI:** 10.21203/rs.3.rs-3035772/v1

**Published:** 2023-07-03

**Authors:** Ken G. Zeng, Tarun Dutt, Jan Witowski, GV Kranthi Kiran, Frank Yeung, Michelle Kim, Jesi Kim, Mitchell Pleasure, Christopher Moczulski, L. Julian Lechuga Lopez, Hao Zhang, Mariam Al Harbi, Farah E. Shamout, Vincent J. Major, Laura Heacock, Linda Moy, Freya Schnabel, Linda M. Pak, Yiqiu Shen, Krzysztof J. Geras

**Affiliations:** 1 New York University, New York, NY, USA; 2 New York University Grossman School of Medicine, New York, NY, USA; 3 New York University Abu Dhabi, Abu Dhabi, United Arab Emirates.; 4 Abu Dhabi Health Services, United Arab Emirates

## Abstract

Pathology reports are considered the gold standard in medical research due to their comprehensive and accurate diagnostic information. Natural language processing (NLP) techniques have been developed to automate information extraction from pathology reports. However, existing studies suffer from two significant limitations. First, they typically frame their tasks as report classification, which restricts the granularity of extracted information. Second, they often fail to generalize to unseen reports due to variations in language, negation, and human error. To overcome these challenges, we propose a BERT (bidirectional encoder representations from transformers) named entity recognition (NER) system to extract key diagnostic elements from pathology reports. We also introduce four data augmentation methods to improve the robustness of our model. Trained and evaluated on 1438 annotated breast pathology reports, acquired from a large medical center in the United States, our BERT model trained with data augmentation achieves an entity F1-score of 0.916 on an internal test set, surpassing the BERT baseline (0.843). We further assessed the model’s generalizability using an external validation dataset from the United Arab Emirates, where our model maintained satisfactory performance (F1-score 0.860). Our findings demonstrate that our NER systems can effectively extract fine-grained information from widely diverse medical reports, offering the potential for large-scale information extraction in a wide range of medical and AI research. We publish our code at https://github.com/nyukat/pathology_extraction.

## Introduction

Artificial intelligence (AI) systems have emerged as valuable tools for handling and making decisions from large amounts of data. Notably, AI systems have demonstrated significant success in detecting and diagnosing breast cancer^1–7^, skin cancer^8,9^, prostate cancer^10,11^, COVID-19^12,13^ and a range of other diseases^14^. These systems utilize a wide range of imaging modalities, including magnetic resonance imaging (MRI)^3^, X-ray^1,2^ and computed tomography (CT) scans^12^ to generate predictions to assist clinicians^1^. However, modern AI systems often require large amounts of labeled data for training and evaluation. Curating these datasets involves extracting relevant annotations from clinical reports which contain vital information regarding a patient’s medical history, including diagnoses, and laboratory results among others. Integrating this information into AI systems can enhance their accuracy and reliability in disease diagnosis and treatment planning. However, manually extracting this information is time-consuming, so a commonly adopted approach to extracting these findings is to use a mixture of rule-based systems with regular expressions^15–17^.

Automatic extraction of information from human-written text is challenging for three primary reasons. Firstly, while rule-based systems are sensitive to minor changes in the input^17,18^, natural language allows for an almost limitless range of expressions that preserve the same meaning. Secondly, negation poses a significant challenge, and hence contextual understanding is often required for this task. Both introduce a large number of possible variations, and existing systems have to memorize and search for a large list of possible terms and counterexamples^16^. Lastly, strictly rule-based systems that depend on a pre-defined list of terms have difficulties dealing with errors such as misspellings or formatting issues^18^. While some advanced rule-based approaches have been developed to address these challenges^19^, research has shown that they require a disproportionate amount of manual labor to account for the subtle nuances of human language^20^.

Statistical machine learning offers an alternative solution that can address some of the limitations of rule-based methods. Existing approaches often formulate pathology extraction as a report classification problem, by limiting the extracted information such as diagnosis to a finite set of possible outcomes^21–25^. However, such systems can only extract a predetermined number of findings from a single report. If a report includes multiple findings, each having distinct characteristics, a report classification model would not be able to extract the relationships between each finding and its specific attributes, such as its position or grade. Additionally, if there is a large variety of possible findings, then the model either needs to lose information or have an increasingly large number of possible categories.

Other approaches frame the report information extraction task as a word classification problem^26,27^. Such systems categorize each word within a particular report into one of many pre-defined entities, such as ‘lesion position’, ‘cancer subtype’, ‘cancer grade’, etc. However, with word classification, a system can achieve reasonable performance by only partially extracting clinically relevant terms. In the case of breast cancer, a system might learn to label a common but incomplete phrase such as ‘carcinoma in situ’. However, this partial phrase is not useful because it could refer either to ‘ductal carcinoma in situ’ (a malignant finding), or ‘lobular carcinoma in situ’ (a high-risk benign finding). Therefore, simply identifying individual keywords is insufficient; instead, it is essential to recognize and group clinically relevant phrases collectively.

Named entity recognition (NER) is a popular NLP technique used to identify and classify entire phrases or entities. Unlike rule-based systems, NER models utilize deep learning algorithms, such as BERT^28^, to learn patterns from human-annotated reports, allowing them to handle a wide range of inputs and adapt to new contexts^29^. However, training these systems requires large, detailed datasets, which are often labor-intensive and costly to collect. Conversely, training with small datasets can result in overfitting and poor generalization. Data augmentation techniques can address these issues by generating synthetic training examples through minor alterations to existing text, creating a larger, diverse dataset that represents real-world reports. By leveraging data augmentation techniques, NER systems can be trained more efficiently, resulting in improved accuracy and performance in identifying and classifying named entities in text data^30^.

**Figure 1.**
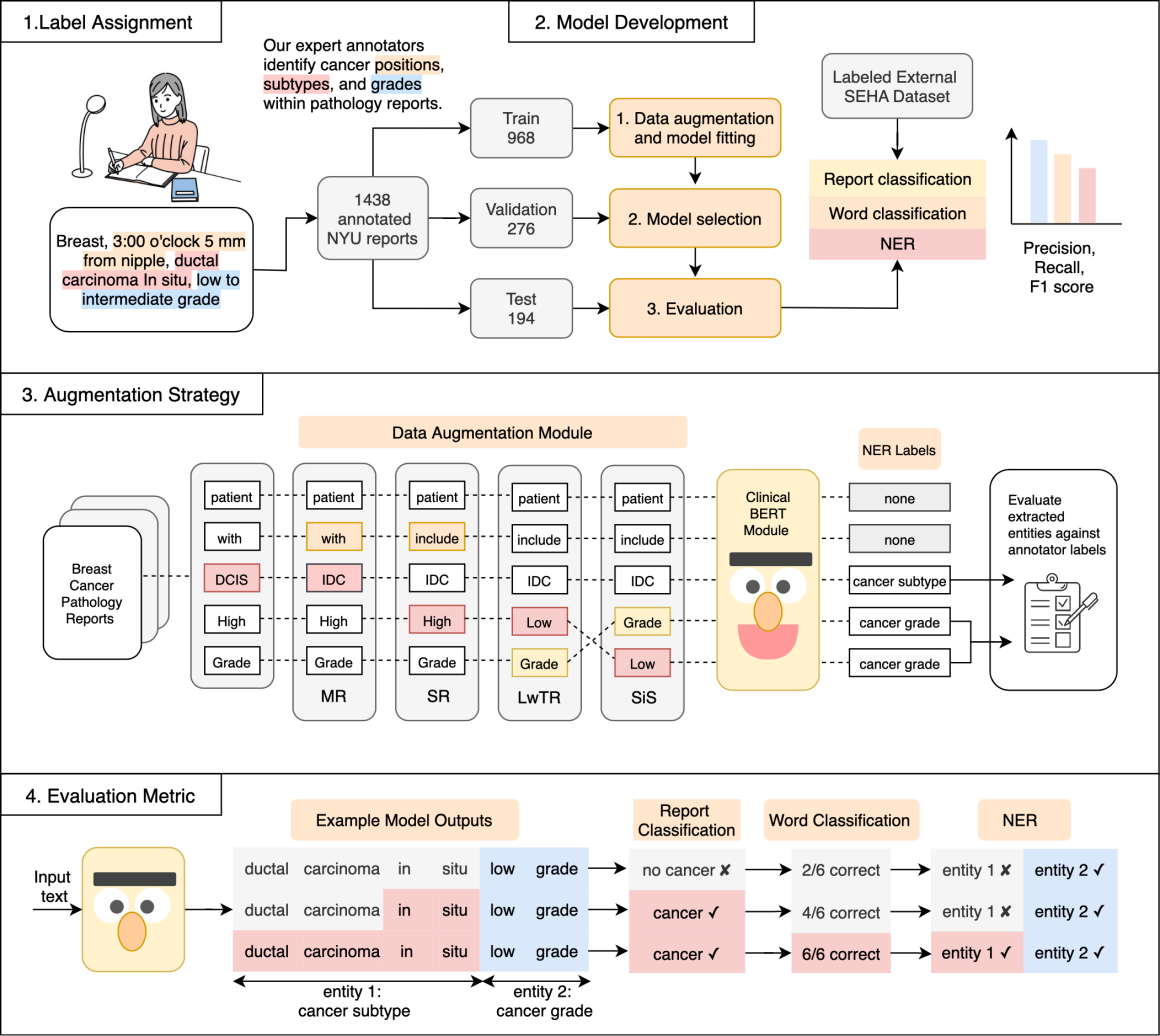
Overview of the proposed NER system. (1) Our annotators identified clinically relevant phrases within each pathology report as ground-truth labels. (2) We collected 1438 reports from NYU Langone Health’s electronic health record database and split them into training, validation, and test sets. (3) To improve robustness, we explored four data augmentation strategies, which are elaborated in detail in Data Augmentation and Preprocessing. (4) We evaluate our approach using three protocols: report classification, word classification, and NER evaluation.

In this work, we present a NER system (see Figure 1) to extract three key elements from breast pathology reports: cancer grade, cancer subtype, and lesion position. We evaluate the effect of data augmentation (see Data Augmentation and Preprocessing for more details on each approach) on our model’s performance against different baselines in three settings, ordered by increasing difficulty: report-level classification, word classification, and entity recognition. We use precision, recall, and F1 scores for each of these protocols to evaluate the model’s accuracy and robustness. Trained and evaluated on 1,438 annotated breast pathology reports from a large U.S. medical center, our model achieves an entity F1-score of 0.916, surpassing a strong BERT baseline (F1-score 0.843). Moreover, we also demonstrate the generalization of the NER system to an unseen external validation dataset from the United Arab Emirates. We also show that our NER model can be adapted for simpler classification tasks, such as report and word classification, without performance degradation.

## Results

### Datasets

The NER system was developed and evaluated using the NYU Breast Pathology Reports dataset consisting of 1438 reports collected from 1350 patients examined between 2018 and 2020 at NYU Langone Health in New York, USA. The NYU Langone Health hospital system spans more than 320 sites across New York City and Long Island, allowing the inclusion of a diverse patient population. We randomly split this dataset into a training set (968 reports) used to optimize the parameters of the model, a validation set (276 reports) used for model selection and hyperparameter tuning, and a hold-out test set (194 reports) used for evaluation.

To assess the NER system’s generalizability, we used an external validation dataset provided by the Abu Dhabi Health Services Company (SEHA) in the United Arab Emirates (UAE) (IRB approvals HRPP-2022-51 and SEHA-IRB-042). SEHA operates all public hospitals and clinics across the emirate of Abu Dhabi. The SEHA dataset consists of 55 reports from 55 unique patients admitted to 11 different hospitals and clinics in Abu Dhabi between 2008 and 2022. The patients’ ages ranged from 15 to 90 years, with a mean age of 42 years. More detailed statistics of our dataset can be found in Table 1.

### Report Classification

In this task, we use our model to generate a single prediction for each report which indicates whether a report contains any cancer-positive findings or not. Cancer-positive findings include primary breast cancers: invasive ductal carcinoma, invasive lobular carcinoma, special-type invasive carcinoma (including tubular, mucinous and cribriform carcinomas), intraductal papillary carcinoma, microinvasive carcinoma, ductal carcinoma in situ, as well as non-primary breast cancers: lymphoma. We formulate this task as a binary prediction (cancer vs. no cancer). We compare our method against a rule-based baseline, XGBoost^31^ classifier using TF-IDF (term frequency − inverse document frequency) features^32^, an LSTM (long short-term memory) model^33^, and a BERT model^34^ (see Baseline Approaches). Experimental results show that the Our model outperforms all baselines for this task (see Table 2). Our model achieves an F1 score of 0.993, while the rule-based baseline, TF-IDF baseline, LSTM baselines, and BERT achieve F1 scores of 0.975, 0.983, 0.989, and 0.972, respectively.

### Word Classification

For word classification, the text is broken down into individual words and each word is given a label. The goal is to correctly predict the class (cancer grade, cancer subtype, lesion position, or other) of each word in the reports. This is a more difficult task than report-level classification, as the word classification model must make predictions for every single word in the report, which introduces a greater potential for error. We compare our model against several baselines (see Baseline Approaches) including LSTM, and BERT (without augmentations). In Table 3, we show that the proposed training augmentation strategies and BERT model significantly improve the benchmark BERT baseline. Our model achieves an F1-score of 0.988, 0.974, and 0.929 on cancer subtype, cancer grade, and lesion position. In comparison, the BERT baseline achieves lower F1-scores of 0.934, 0.959, and 0.866, respectively. However, our analysis also reveals that LSTM attains higher precision than our approach for cancer subtype and position, albeit with a notably lower recall.

### Entity Recognition

We examine the performance of our model for entity recognition, a task that requires exact sequence alignment, which is more rigorous than word classification. In Table 4, we also show that using all four augmentation techniques improves the BERT’s performance, leading to an overall weighted F1 score of 0.916. Further analysis reveals that the high word-level precision of the LSTM model is offset by its poor entity-level performance, indicating that it can effectively memorize keywords within relevant entities but struggles to identify complete entities. In contrast, our approach achieves an F1 score of 0.983 for cancer grade, 0.928 for cancer subtype, and 0.827 for position, compared to the baseline scores of 0.873, 0.903, and 0.706, respectively. This demonstrates that our augmentation strategies effectively improve the BERT’s generalization ability on unseen test data.

### External Validation

In this study, we assessed the performance of our NER model on an external dataset comprising 55 breast pathology reports from SEHA in Abu Dhabi, UAE. The model’s performance is presented in Table 5. Although the baseline BERT model can effectively parse reports in our internal database, its performance declines substantially when encountering shifts in the underlying structure. In Error analysis, we provided some examples and explanations to illustrate why this occurs. Nevertheless, our data augmentation policy enhances the model’s resilience to such shifts, as it outperforms the baseline in all three extracted categories.

### Error analysis

In Table 6, we compare our model’s prediction against the baseline approach to understanding the strengths and weaknesses of our method. In the first example, we show a case that the baseline incorrectly identified the word ‘ductal’ as a cancer subtype, since this word appears often in valid cancer subtypes. In the third example, our model utilizes the surrounding context and correctly identifies that the mention of “ductal carcinoma in situ” is meant as negation while the baseline fails to do so. In fact, although the baseline LSTM approaches produce a high word-level classification performance, they often fail to identify the entire entity correctly.

In Table 7, we identified several factors in clinical settings that presented significant challenges. In the third example from the internal NYU dataset, the administration of chemotherapy treatment prior to excision led to the variability of the language used in the pathology report. The variability in biopsy types, including fine needle aspirations, made it challenging to differentiate between ductal carcinoma in situ (DCIS) and papillary carcinoma due to insufficient tissue. These challenges were further magnified in the external dataset that contained language that the model was not previously exposed to (positions such as ‘periareolar’ and ‘uoq’), resulting in high precision but low recall.

In Table 7, we provided additional examples where our model fails on the external SEHA dataset. The reports in the external dataset are generally less structured and standardized than our internal test set. As an example, “focally high-grade DCIS” would typically be written as “focal ductal carcinoma in situ, high grade.” Additionally, we present examples to illustrate how writing conventions differ between the two datasets. In the fourth example of the SEHA dataset, although the report lists “ductal carcinoma in situ,” it immediately writes that no DCIS is present, making it a confusing instance of negation. Despite these challenges, our data augmentation strategies enhance our model’s resilience against format changes.

## Discussion

In this study, we were able to develop a machine learning approach that effectively extracted clinical entities from pathology reports and demonstrated significant improvement when compared to previous benchmarks. Our approach adapts upon a flexible clinical BERT model^34^ that was extensively pre-trained on electronic health records and avoids the costly training of a new language model. Furthermore, we fine-tuned our model for the NER task, offering a flexible and scalable solution for many applications^35^.

While TF-IDF^32^ and LSTM^33^ have been successful in text classification, large language models (LLM) such as BERT have produced state-of-the-art performances on more complex tasks such as NER^36,37^. One of the key advantages of BERT over other approaches is its ability to capture the context and meaning of words in a sentence, by considering the surrounding contexts of each word and understanding the nuances of natural language. Our algorithm is designed to accurately and efficiently extract the sub-type, cancer grade, and lesion position information. By automating this process, our algorithm significantly reduces the time and effort required for manual information extraction, enabling researchers to analyze a larger volume of data in a more timely and efficient manner.

Our research demonstrates that data augmentation significantly improves the robustness of our model, and we identify two main reasons for this. Firstly, by adding noise to the training data, the model is exposed to previously unseen combinations of text, which approximates the variability and error that humans encounter. This helps prevent the model from simply memorizing the same cases and instead encourages it to learn more general patterns. Secondly, augmentations that modify key entities make the keywords themselves less reliable during training, which prompts the model to rely more heavily on contextual information and improve its performance on unseen test sets. Our approach was particularly effective, allowing us to achieve strong performance with a relatively small training dataset.

In addition, we tested our approach on an external dataset and demonstrated its effectiveness from a wide range of breast cancer pathology reports. Our machine learning approach is adaptable and the system will remain viable and sustainable for the foreseeable future. While the initial F1 scores may be impacted by variations in report formats and language use across institutions, our system’s ability to perform consistently and accurately on an external dataset shows its robustness and reliability, making it a valuable tool for breast cancer research.

We recognize that our system has a number of limitations. Despite our approach’s effectiveness, it requires a considerable amount of manual labeling. Deep learning models, being more complex than rule-based systems, present challenges in troubleshooting the cause of failures. These models often involve numerous interconnected layers and millions of parameters, making it challenging to understand why a particular decision was made. In contrast, rule-based systems rely on explicitly defined rules, making it easier to trace back decisions and identify the cause of errors. Furthermore, although our current augmentation techniques offer a cost-effective alternative to creating synthetic training data, examples generated by these augmentation strategies may contain grammatical errors and unnatural language. Recent studies^38^ have shown that large-scale language models can generate artificial training data to overcome these limitations, which could improve the accuracy and robustness of our approach. Additionally, expanding the training set would also allow us to fine-tune the approach to identify additional entity categories, such as tumor size, tumor biomarkers, and genetic testing. The extensive tumor profile could be automatically synthesized into a standardized format and integrated into the clinical workflow, streamlining the reporting process and facilitating data analysis.

A major issue in machine learning for medicine is that most current clinical information extraction systems are tailored to each institution’s unique requirements. Therefore, such systems frequently fail to generalize to data from new sources, hindering collaboration between different institutions^35^. Our work suggests that there is room for research into potentially generalizable clinical information systems, such as auto-regressive models such as GPT^39^ and its derivatives. These large models can learn clinical tasks with only a handful of training examples^40,41^. Moreover, recent developments in NLP have demonstrated that human feedback can significantly improve AI systems^42^, suggesting that reinforcement learning could be a potential area of research.

## Methods

This study, approved by the NYU Langone Health Institutional Review Board (IRB) under the reference number # i18-00712_CR3, was deemed compliant with the Health Insurance Portability and Accountability Act (HIPAA). Informed consent was waived by the IRB for this study. All methods employed in the study were conducted in strict accordance with the relevant guidelines and regulations.

### Data annotation

All breast pathology reports were reviewed by three fourth-year medical candidates who have completed their core clerkships and one radiology resident who has completed their breast radiology rotation. Each annotator recorded the start and end indices of all phrases belonging to the cancer subtype, cancer grade, and lesion entity types. Text annotations were translated to word-level labels in the IO scheme^43^. In this labeling scheme, labels are assigned to each word token within the dataset. Words that correspond to cancer subtype, cancer grade, and lesion position were labeled ‘I-<entity type>‘, while everything else is labeled ‘O’ (others).

### NER Formulation

In this section, we provide a mathematical formulation of our NER task. Given a sequence $x=\left(x_{1},\ldots x_{m}\right)$ consisting of m words, the goal of NER is to predict a list of tuples ($I_{s}$, $I_{e}$, c), where $I_{s}$ and $I_{e}$ denote the starting and ending index of the entity and c∈C={cancersubtype,cancergrade,lesionposition,other} denotes the entity category^44^.

### Transformer Model

Pretrained transformer models such as BERT^28^ achieved success in a wide range of NLP tasks. They allow the AI systems to capture complex and long-term interactions between different input words better than the recurrent neural networks^45^. BERT uses a self-attention mechanism, which allows it to attend to both past and future tokens in the input sequence. In our work, we use BioBERT trained on clinical notes, which we will refer to as clinical BERT, as a base model for fine tuning^34^. Empirically, clinical BERT is shown to have improved performances on multiple downstream clinical tasks, when compared with the base BioBERT and base BERT models^34^. Additionally, BERT has been shown to be capable of identifying negation^46^ and correcting misspellings^47^.

To use BERT for NER, we first tokenize relevant text into word tokens, which are further broken down into sub-word tokens using the WordPiece tokenizer^48^. These tokens are converted to BERT representations vectors, serving as inputs for the fully connected output classifier. The output classifier consists of a feed-forward network with a softmax layer which produces a probability of assigning each entity category to an input token. The model outputs corresponding to the first subtoken in each word are used as the word-level predictions, while the model outputs for the remaining tokens are ignored.

We train our model to predict the class of each word token $y=\left(y_{1},\ldots ,y_{m}\right)$ from $x=\left(x_{1},\ldots x_{m}\right)$, where $y_{i}\in C$ is the entity category. We first convert each input word into a hidden vector $h\in R^{H}$ using a BERT model. Let $W\in R^{|C|\times H}$ denote the learnable parameters of the fully connected layer. Then we calculate scores $z_{i}\in R^{\left|C\right|}:z_{i}=W\cdot h_{i}$, which indicate the confidence of the model in assigning each class to this input token $x_{i}$. We then convert these scores to probabilities using the softmax function: $p\left(y_{i}=c|z_{i}\right)=softmax{\left(z_{i}\right)}_{c}$. Let $X=\left(x^{\left(1\right)},\ldots x^{\left(N\right)}\right)$, $Y=\left(y^{\left(1\right)},\ldots y^{\left(N\right)}\right)$ represent the input data, where each $x_{j}^{\left(i\right)}$ represents *j*th token in the *i*th report. We add padding tokens to the end of all input sequences to ensure that all inputs have the same length of 128. The padding tokens are ignored while training and do not contribute to the loss. Using the data, we trained such a system using the Pytorch^49^ implementation of the cross entropy loss function:

$$l(Y,X)=\sum_{i=1}^{N}\;\sum_{j=1}^{m}\;log\left(\frac{{exp}_{j,y_{j}^{\left(i\right)}}^{\left(i\right)}}{\sum_{c=0}^{C}expz_{j,c}^{\left(i\right)}}\right)\cdot I(y_{j}^{\left(i\right)}\neq \text{ignored})$$

### Baseline Approaches

To evaluate the performance of our approach and determine its strengths and weaknesses, we conducted a comparative analysis with several commonly used existing approaches in the field.

#### Rule-based Approach

The rule-based method relies on string matching to identify clinically relevant phrases. This method collects a comprehensive list of lexicons about malignant findings and matches them against the text. This approach has limitations, especially when dealing with text that has minor changes, as it requires the system to look for a variety of patterns to match a single word, such as “.eft”, “l.ft”, “le.t”, and “lef.” (where ‘.’ can match any non-newline token). Additionally, this method can only determine the presence or absence of malignant findings, rather than identifying the full extent of malignant findings. This limitation makes the method much more limited in clinical practice. More details about this rule-based approach can be found in this technical report^16^.

#### XGBoost

We use a TF-IDF vectorizer to convert the report into a numerical vector. TF-IDF is a word count technique used to assign numerical weights to words in a document, based on how frequently they appear in the document and how important they are to a corpus^32^. XGBoost is a gradient-boosting machine learning library that uses decision trees as its base learner and achieves state-of-the-art performances in tabular classification problems^31^. The XGBoost model takes in the numerical vectors as input and outputs a predicted label based on the learned decision boundaries between the different classes, such as the presence or absence of any cancer findings within a pathology report. Such systems have been used for clinical report classification, such as identifying drug mechanisms^50^ or identifying genetic mutations^51^.

#### LSTM

LSTM^33^ is a type of recurrent neural network that is widely used for NLP tasks, including NER^52,53^. The LSTM model has several layers of memory cells that enable it to remember information from previous time steps and selectively forget information when needed. LSTM uses fixed-length vector representations for each word, which can capture a limited context of the entire input sequence. This ability to capture contextual information is essential for NER, where the meaning of a word can depend on its surrounding words. Additionally, LSTM models typically require significant amounts of task-specific training data to achieve high performance, which limits its effectiveness given our dataset size.

### Data Augmentation and Preprocessing

In our work, we used the following data augmentation transformations: mention replacement (MR), synonym replacement (SR), label-wise token replacement (LwTR), and shuffle within segments (SiS). For each augmentation, we draw a sample from a Bernoulli distribution to decide whether we apply the augmentation to each instance. For our use case, we experimented with probability out of 0.05, 0.1 and 0.15. Mention replacement splits the text into segments, where each segment contains a sequence of characters with the same entity label. Each segment then has a random chance of being replaced by another sequence in the dataset with the same label. Synonym replacement randomly replaces each word with a synonym from Wordnet^54^, a lexical database for the English language. Label-wise token replacement randomly replaces each word token in the text with another word from the dataset that has the same entity label. Shuffle within segments randomly shuffles the order of tokens within each entity segment in the text. While this approach may produce incoherent text, prior work has shown that data augmentation leads to an improvement in NER performance when used with large pre-trained transformers^30^. To enhance the robustness of our data analysis, we employed the four augmentation strategies, namely MR, SR, LwTR, and SiS, in this specific order to ensure consistency and accuracy in our results.

### Evaluation

All our models, including XGBoost baselines, BERT baselines, LSTM baselines, and BERT models with augmentation, generate a probability distribution of the class (cancer subtype, cancer grade, lesion position, and others) for each word. To circumvent the complexity of selecting thresholds, we assign the class with the highest probability as the prediction for each word. Subsequently, we compare these predictions with the ground-truth labels using NER sequence evaluation metrics commonly employed in NLP benchmarks^55,56^. We define an entity as predicted correctly if all of the words between the start and end tokens are predicted accurately. We then use the weighted sum of the precision, recall, and F1 scores of all the entity classes as performance metrics to evaluate our NER system.

### Training

We initialized our model parameters with the pre-trained clinical BERT model parameters from Hugging Face^57^, with a maximum sequence size of 128 subword tokens. Then we used ADAM^58^ with a cross-entropy loss function to fine-tune the model parameters. We experimented using learning rates 2e-4, 1e-4, 9e-5, 8e-5, and 6e-5. We also experimented with batch sizes of 8, 16, and 32. Finally, we employ different combinations of data augmentation strategies each with a probability of 0.05 or 0.1, 0.15. For early stopping, we selected the model parameters with the lowest validation loss. The optimal model is trained with a batch size of 8, a learning rate of 6e-05, and applying all four data augmentation techniques with a probability of 0.1.

## Figures and Tables

**Table 1.** Number of unique entities and words within the pathology report dataset for each class. Classes such as position contain a large percentage of unique entities, which makes them difficult to capture using rule-based approaches. Cancer grade, in comparison, has far fewer possible options which makes it easier for our model to extract.

Dataset	Category	Total words	Unique words	Total entities	Unique entities

NYU	non-entity	138,083	3,853	-	-
	cancer subtype	4,785	121	1,547	133
	cancer grade	2,783	55	1,026	78
	position	4,117	175	1,063	516

SEHA	non-entity	9,356	929	-	-
	cancer grade	197	13	20	11
	cancer subtype	55	13	65	12
	position	168	47	43	39

**Table 2.** Report classification performance. Applying all available augmentation, which is referred to as ‘BERT + MR-SR-LwTR-SiS’ (see Data Augmentation and Preprocessing) achieved a higher F1 score than baseline approaches. The best results as well as the approach with the highest overall performance are shown in bold. Precision and recall are referred to as ‘pr’ and ‘rec’.

	pr	rec	F1

rule-based	**1.000**	0.951	0.975
TF-IDF + XGBoost	0.967	**1.000**	0.983
LSTM	0.976	0.991	0.989

BERT	0.976	0.971	0.972
**BERT + MR-SR-LwTR-SìS**	0.993	0.993	**0.993**

**Table 3.** Comparing word classification performance between benchmark LSTM and XGBoost models against BERT with different combinations of augmentation policies including MR, SR, lwTR, and SiS. Again, applying all augmentations, which is referred to as ‘+ MR-SR-LwTR-SiS’, produced the best overall performance across all three categories. While LSTM has a higher precision for all entity classes, which gives the impression that the performance is comparable. However, it has a lower recall F1 score which shows that BERT is more suitable for this task.

	cancer grade			cancer subtype			position		
	pr	rec	F1	pr	rec	F1	pr	rec	F1

LSTM	0.942	0.755	0.836	**0.970**	0.767	0.857	**0.914**	0.803	0.855
BERT	0.897	0.973	0.934	0.940	0.979	0.959	0.803	0.940	0.866
+ MR	0.907	0.987	0.945	0.884	**0.984**	0.931	0.894	0.822	0.857
+ MR-SR	0.920	**0.989**	0.953	0.943	0.958	0.950	0.794	0.925	0.854
+ MR-SR-LwTR	0.973	0.979	0.976	0.952	0.975	0.964	0.880	0.915	0.897
**+ MR-SR-LwTR-SiS**	**0.994**	0.982	**0.988**	0.968	0.980	**0.974**	0.898	**0.962**	**0.929**

**Table 4.** NER performance of the LSTM baseline and our model. The best-performing approach for each class is highlighted in bold. The results first show that LSTM performs worse than the fine-tuned clinical BERT model baseline. Second, applying all available augmentation to BERT (referred to as ‘+ MR-SR-LwTR-SiS’) leads to improved performance across all entity classes.

	cancer grade			cancer subtype			position			weighted avg		
	Pr	rec	F1	Pr	rec	F1	Pr	rec	F1	Pr	rec	F1

LSTM	0.887	0.902	0.895	0.729	0.701	0.715	0.741	0.592	0.659	0.807	0.694	0.743
BERT	0.851	0.897	0.873	0.897	0.909	0.903	0.620	0.820	0.706	0.811	0.882	0.843
+ MR	0.879	0.922	0.900	0.842	0.908	0.874	0.772	0.743	0.757	0.829	0.857	0.843
+ MR-SR	0.891	0.939	0.914	0.880	0.873	0.877	0.576	0.803	0.671	0.808	0.876	0.837
+ MR-SR-LwTR	0.954	0.954	0.954	0.906	**0.926**	0.916	0.772	0.850	0.809	0.881	0.912	0.896
**+ MR-SR-LwTR-SiS**	**0.989**	**0.977**	**0.983**	**0.932**	0.924	**0.928**	**0.777**	**0.883**	**0.827**	**0.905**	**0.929**	**0.916**

**Table 5.** NER performance on the external validation set. The augmented BERT’s superior F1 score, in comparison to the baseline BERT, is a testament to the effectiveness of our augmentation policy in enhancing the model’s ability to generalize to unseen texts.

	cancer grade			cancer subtyPe			Position			weighted avg		
	Pr	rec	F1	Pr	rec	F1	Pr	rec	F1	pr	rec	F1

BERT	0.706	0.571	0.632	0.864	0.838	0.851	0.667	0.578	0.619	0.773	0.709	0.739
**+ MR-SR-LwTR-SiS**	**0.864**	**0.950**	**0.905**	**0.882**	**0.923**	**0.902**	**0.786**	**0.767**	**0.776**	**0.847**	**0.875**	**0.860**

**Table 6.** Examples where our model generated the correct prediction but the BERT baseline failed. Predicted entities are colored, purple indicates cancer subtypes, while orange represents cancer grades.

True Entity	Our Prediction	Baseline LSTM Prediction
invasive mammary carcinoma with ductal and lobular fetures	invasive mammary carcinoma with ductal and lobular fetures	invasive mammary carcinoma with ductal and lobular fetures
showing ductal proliferation, which fall short of the criteria for ductal carcinoma in situ	showing ductal proliferation, which fall short of the criteria for ductal carcinoma in situ	showing ductal proliferation, which fall short of the criteria for ductal carcinoma in situ
- ductal caricinoma in situ, low to intermediate nuclear grade, cribriform, papillary and micropapillary types	- ductal caricinoma in situ, low to intermediate nuclear grade, cribriform, papillary and micropapillary types	- ductal caricinoma in situ, low to intermediate nuclear grade, cribriform, papillary and micropapillary types
b. breast, left, segmental excision : - microinvasive ductal carcinoma (see comment)	b. breast, left, segmental excision : - microinvasive ductal carcinoma (see comment)	b. breast, left, segmental excision : - microinvasive ductal carcinoma (see comment)

**Table 7.** Examples where our model generated a different output from the labelers for (1) our internal dataset and (2) the external SEHA dataset, with comments explaining what the error is and why it might have occurred. Predicted entities are colored, purple indicates cancer subtypes, orange represents cancer grades, and teal is used to indicate position.

**Internal NYU Dataset**		
True entity	Our prediction	Comments
left axilla : core biopsy - invasive poorly differentiated carcinoma, high nuclear grade	left axilla : core biopsy - invasive poorly differentiated carcinoma, high nuclear grade	Our model disagrees with labelers for cases where the cancer grade is within the cancer subtype
related changes size : 3.5 cm location : 12 to 2 o’clock, margins: not involved	related changes size : 3.5 cm location : 12 to 2 o’clock, margins: not involved	Our model can fail to include key terms
saw pattern is noted - papillary carcinoma, at least ductal carcinoma in situ (dcis), with solid, papillary, focal cribriform	saw pattern is noted - papillary carcinoma, at least ductal carcinoma in situ (dcis), with solid, papillary, focal cribriform	The model believes the two entities mentioned refer to the same lesion, while the labelers treated them as separate
		
**External SEHA Dataset**		
True entity	Our prediction	Comments
- dcis - focally high grade dcis with apocrine changes present. - ER: positive,	- dcis - focally high grade dcis with apocrine changes present. - ER: positive,	The term ‘dcis’ is generally written in full for our dataset
b - right breast core biopsy 10/2 cm from the nipple	b - right breast core biopsy 10/2 cm from the nipple	10/2 cm would generally be written as ‘10 o’clock 2 cm’ in our dataset
core biopsy right breast uoq, consult material from national reference laboratory	core biopsy right breast upper outer quadrant, consult material from national reference laboratory	‘uoq’ is generally written in full as ‘upper outer quadrant’ in our dataset
ductal carcinoma in situ (dcis): no dcis is present macroscopic and microscopic extent of tumor skin	ductal carcinoma in situ (dcis): no dcis is present macroscopic and microscopic extent of tumor skin	The way that negation is expressed can be confusing for our model to identify accurately

## Data Availability

Although we cannot make the dataset public, we will evaluate models from other research institutions on the test part of the data set upon request. For any further queries regarding data availability, please contact Krzysztof J. Geras (k.j.geras@nyu.edu).
